# Older adults during the pandemic: Mental health symptoms are predicted by childhood trauma

**DOI:** 10.1192/j.eurpsy.2022.778

**Published:** 2022-09-01

**Authors:** V. Békés, J.C. Perry, C. Starrs

**Affiliations:** 1 Yeshiva University, Ferkauf Graduate School Of Psychology, Bronx, United States of America; 2 McGill University, Psychiatry, Montreal, Canada; 3 CUNY Postdam, Psychology, Potsdam, United States of America

**Keywords:** Covid-19, Childhood Trauma, Older Adults, Distress

## Abstract

**Introduction:**

It has been broadly anticipated that COVID-19 pandemic-related experiences may constitute traumatic stressors in vulnerable populations, and that older adults’ might be especially at risk of experiencing mental health symptoms during the pandemic.

**Objectives:**

The present study aimed to examine older adults’ psychological distress: posttraumatic stress, Covid-related fears, anxiety, and depression during the pandemic, and the relationship between present distress, defensive functioning, and childhood trauma. We also explored potential differences between *younger-older adults* (between 65 and 74 years), and *older-older adults* (75 years and above).

**Methods:**

Data was collected in a large-scale online survey during the early months of the pandemic, for the present study, we included participants above 65 years old (*N* = 1,225).

**Results:**

showed that age, adverse childhood experiences, and overall defensive functioning were all significantly related to posttraumatic stress, anxiety, and depression. Specifically, younger age and more reported childhood adversity were related to higher distress, whereas higher defensive functioning was related to less distress. Covid-related fears were not associated with age. Our final model showed that defensive functioning mediated the relationship between childhood trauma and distress.

**Conclusions:**

Our results support the relative resilience of older-older adults compared to younger-older adults, as well as the long-lasting impact of childhood adversity through defensive functioning later in life, specifically in times of heightened stress, such as the COVID-19 pandemic. Future studies are warranted to identify further factors affecting defensive functioning as adults age, as well as processes that are associated with resilience in response to stressors in older adulthood.

**Disclosure:**

No significant relationships.

